# Multicentre phase II study of bifractionated CPT-11 with bimonthly leucovorin and 5-fluorouracil in patients with metastatic colorectal cancer pretreated with FOLFOX

**DOI:** 10.1038/sj.bjc.6602194

**Published:** 2004-10-05

**Authors:** F Recchia, G Saggio, A Nuzzo, A Lalli, L Di Lullo, A Cesta, S Rea

**Affiliations:** 1 Unità operativa di Oncologia, Ospedale Civile di Avezzano, Italy; 2 Unità operativa di Oncologia, Ospedale Civile di Lanciano, Italy; 3 Unità operativa di Oncologia, Ospedale Civile di Teramo, Italy; 4 Unità operativa di Oncologia, Ospedale Civile di Isernia, Italy; 5 Università degli studi de L'Aquila, Italy; 6 Fondazione ‘Carlo Ferri’, Monterotondo, Roma, Italy

**Keywords:** CPT-11, 5-fluorouracil, leucovorin, second-line chemotherapy

## Abstract

This multicentre phase II study was designed to evaluate the antitumour activity and toxicity of bifractionated camptothecin (CPT-11) and 5-fluorouracil/ leucovorin (5-FU/LV) in the treatment of patients with metastatic colorectal cancer (MCC) who had been pretreated with 5-FU/LV-oxaliplatin (FOLFOX regimen). In all, 35 patients were enrolled in a two-stage trial. Treatment consisted of two daily doses of CPT-11, 90 mg m^2^ administered over 90 min, followed by LV, 200 mg m^2^ administered over 2 h plus 5-FU 400 mg m^2^ as a bolus and 600 mg m^2^ as a 22-h continuous infusion administered with disposable pumps as outpatient therapy. Toxicity was closely monitored. Response was evaluated by computed tomography scans every 8 weeks. All 35 patients were assessable for toxicity and response to treatment. Seven patients had a partial response, giving an overall response rate of 20%; 11 patients had stable disease (31.4%) and 17 progressed (48.5%). The median progression-free survival was 7.1 months and median survival was 14 months. A total of 10 patients (30%) experienced grade 3–4 toxicity, including nausea (15%), diarrhoea (12%) and neutropenia (15%), while seven patients (21%) had grade 2 alopecia. The bifractionated bimonthly schedule of CPT-11 plus 5-FU/LV showed substantial antitumour activity and was well tolerated in this group of patients with a poor prognosis, pretreated with the FOLFOX regimen.

Colorectal carcinoma, the second most common cancer in Europe, accounts for 80 000 to 95 000 deaths each year (Black *et al*, 1997). Systemic chemotherapy has gained a key role in the treatment of colorectal cancer: in the adjuvant setting it decreases the chance of recurrence and improves survival in patients with node-positive tumours (O'Connell *et al*, 1997), whereas in the setting of metastatic disease it delays the onset of tumour-related symptoms and extends survival (Nordic Gastrointestinal Tumour Adjuvant Therapy Group, 1992). Two recently introduced drugs active in the treatment of MCC are the camptothecin analog irinotecan (CPT-11) and oxaliplatin (L-OHP). Owing to the fact that these drugs have completely different mechanisms of action, they do not present crossresistance: CPT-11 inhibits cell division by inactivation of topoisomerase I (Jaxel *et al*, 1989) and is non-crossresistant with 5-fluorouracil (5-FU), while L-OHP forms DNA adducts leading to the inhibition of DNA synthesis (Raymond *et al*, 1998). Both drugs have been shown, *in vitro*, to have synergistic effects with 5-FU and leucovorin (LV) on colorectal cancer cell lines (Mullany *et al*, 1998; Raymond *et al*, 1998). In Europe, L-OHP combined with LV and infusional 5-FU was approved in 1999 as the first-line treatment of metastatic colorectal cancer (MCC) (de Gramont *et al*, 2000), whereas in North America, a combination of CPT-11 with 5-FU and LV administered as an intravenous (i.v.) bolus (Saltz *et al*, 2000) has been adopted. The FOLFOX regimen (L-OHP and infusional 5-FU plus LV) has recently been reported to be active and comparatively safe and is now recommended as the standard therapy for patients with advanced colorectal cancer (Goldberg *et al*, 2004). In second-line therapy, the association of L-OHP with 5-FU/LV in the treatment of patients with MCC progressing after CPT-11/5-FU/LV (FOLFIRI regimen) has been shown to be beneficial (Rothenberg *et al*, 2003) and limited data from literature exist on the salvage treatment of patients with MCC progressing after the FOLFOX regimen. In patients with resistance to 5-FU bolus, CPT-11 has been shown to be superior both with respect to best supportive care and to 5-FU continuous infusion (Cunningham *et al*, 1998; Rougier *et al*, 1998). A modest survival gain was obtained with this treatment, but was accompanied by severe gastrointestinal toxicity, with an elevated percentage of patients having to be hospitalised during the course of chemotherapy. Such high toxicity has decreased the potentially universal adoption of CPT-11 in the treatment of MCC (O'Connell, 1998).

It has been shown that CPT-11 efficacy and toxicity are both schedule and dose dependent (Albigerges *et al*, 1995; Guichard *et al*, 1997). In a previous study in a group of 54 patients with MCC, the dose of CPT-11, administered as first-line chemotherapy, was split over 2 days and administered with the ‘de Gramont’ regimen in order to decrease the toxicity profile and to better exploit the synergistic action of CPT-11, 5-FU and LV (Recchia *et al*, 2003). As a modest toxicity profile with an activity comparable to other CPT-11-based regimens was observed, in the present study, we have treated a cohort of patients with MCC progressing after the FOLFOX regimen, with bifractionated CPT-11 and bimonthly L-OHP and 5-FU.

## PATIENTS AND METHODS

### Eligibility criteria

Patients previously treated with the FOLFOX regimen for metastatic disease were enrolled in the study. Disease progression had to have occurred during or within 6 months after L-OHP/5-FU/LV-based chemotherapy for metastatic disease. Patients had to be at least 18 years old and ambulatory, with an Eastern Cooperative Oncology Group (ECOG) performance status (PS) ⩽2. The study included only patients with a life expectancy of at least 12 weeks and adequate haematological reserve and hepatic and renal function, documented by WBC ⩾3000 mm^3^, absolute neutrophil count ⩾1500 mm^3^, haemoglobin level >9.0 g dl, platelets ⩾100 000 mm^3^, serum bilirubin ⩾1.5 mg dl, aspartate aminotransferase and alanine aminotransferase <4 times the upper limit of normal) and normal cardiac and renal functions (ejection fraction >50%, serum creatinine ⩽2.0 mg dl).

Patients with additional malignancies, other than curatively treated skin and cervical cancer or with active cardiovascular disease, were excluded. Patients treated with palliative radiation therapy were entered if previous treatment did not involve the lesion used for the measurement of response. The protocol was approved by the Ethical Committee of the Civilian Hospital of Avezzano, Italy and of the other participating institutions, and written informed consent was obtained from each patient.

### Chemotherapy

Following an initial assessment, a single lumen Hickman line or a port-a-cath was positioned into the subclavian vein under local anaesthesia. Patients were instructed in catheter care and heparin flush technique. Chemotherapy was administered on an outpatient basis for 2 consecutive days and was repeated every 2 weeks until disease progression, unacceptable toxicity or refusal. According to *in vitro* studies that had shown that the schedule of administration was a critical parameter for chemotherapeutic efficacy (Guichard *et al*, 1997), CPT-11 was given as the first drug at the dose of 90 mg m^−2^ in 250 ml of 5% dextrose in water over 90 min. Atropine and loperamide were used according to the manufacturer's guidelines. LV 200 mg m^−2^ was administered as a 2-h i.v. infusion, followed by 5-FU 400 mg m^−2^ as a bolus; 5-FU 600 mg m^−2^ was administered as a 22-h continuous infusion over 2 consecutive days with elastomeric pumps. Routine antihemetic prophylaxis with a 5-hydroxytryptamine-3 receptor antagonist was carried out.

Patients were assessed for toxicity before each cycle of chemotherapy using WHO criteria (Miller *et al*, 1981). CPT-11 dosage was modified according to the level of toxicity occurring during the previous course of chemotherapy.

### Pretreatment and follow-up evaluation

Before treatment, a complete history was taken and a physical examination was performed. Weight was recorded and a complete blood count, differential, serum bilirubin, creatinine, albumin, alkaline phosphatase, transaminases, lactic dehydrogenase and carcinoembryonic antigen (CEA) were determined. Initial radiological investigations included chest X-ray and computed tomography of the abdomen and pelvis. Blood counts were repeated weekly, serum biochemistry was determined before each course of treatment and CEA and radiological investigations were repeated every 8 weeks (four courses of chemotherapy) or sooner, if clinically indicated. An X-ray skeletal survey was performed when abnormal areas of uptake were observed in bone scans; CT scanning was used to evaluate hepatic lesions. Before each subsequent course of treatment, all patients had a further complete blood cell count, plasma urea, electrolytes, serum creatinine and liver function tests. In addition, a full blood count was repeated weekly. Follow-up visits were performed bimonthly. Objective responses and toxicity were evaluated according to WHO criteria (Miller *et al*, 1981).

Dose intensity (DI), calculated according to the Hryniuk method (Hryniuk, 1988), was considered as the number of milligrams of the drug per square meter per week during treatment from day 1 of the first cycle to day 15 of the last course of chemotherapy. Planned DI was 90 mg m^2^ per week for CPT-11, 200 mg m^2^ for LV and 1000 mg m^2^ for 5-FU.

### Statistical considerations

The study was designed as a two-stage trial with an interim analysis after treatment of the first group of 18 patients (Simon, 1989). A response rate of >10% was required for the trial to continue. Taking into consideration a 90% response detection rate, in a cohort of 18 evaluable patients, the trial would have terminated if there were ⩽2 responses. As there were >3 partial responses, the trial continued with the enrolment of an additional 17 patients for a total of 35 patients. The primary end points of the study were response rate and toxicity; secondary end points were time to progression and survival. For the response rate, exact binomial 95% confidence intervals (CI) were calculated. Time to progression was measured from the date of the first course of treatment to the date of relapse or last follow-up. Survival was determined from the date of the first course of treatment to the date of death, or 31 December 2003 for surviving patients. Both were assessed by means of the Kaplan and Meier product-limit method (Kaplan and Meier, 1958). The overall survival and toxicity results are presented on an intent-to-treat basis.

## RESULTS

### Patients' characteristics

From January 2000 to December 2002, 35 patients with MCC were enrolled into the trial. Patient's characteristics and history of disease are summarised in Table 1

**Table 1:** Characteristics of patients

**Characteristics**	**No.**	**%**
No of patients	35	100

*Age (years)*		
Median	55	
Range	31–79	
*Sex*		
Males	26	74
Females	9	26
*Performance status (ECOG)*		
0–1	29	83
2	6	17
		
*Site of primary disease*		
Colon	26	74
Rectum	9	26
		
*Stage at diagnosis*		
IIB	3	8
IIIB	2	6
IIIC	7	20
IV	23	66
		
*Prior therapy*		
Surgery	30	86
Radiotherapy	9	26
Adjuvant chemotherapy	10	28
		
*No of previous chemotherapy*		
First line	16	46
Second line	16	46
⩾Third line	3	8
		
*Metastatic sites*		
Liver	25	71
Lung	10	29
Nodes	14	40
Bones	3	9
Pelvic local recurrence	6	17
Peritoneum	8	23
Rising serum markers	1	3
		
*No of metastatic sites*		
1	18	51
2	13	37
⩾3	4	11

ECOG=Eastern Cooperative Oncology Group.

. The median age was 55 years (range 31–79 years) and 74% of patients were males. In all, 74% of patients had colon carcinoma, while 26% had rectal carcinoma. All the patients were pretreated with the FOLFOX regimen for metastatic disease and had received a median number of eight courses of such therapy. A total of 25 patients (71%) had liver metastases, 29% had lung disease and 40% had nodal involvement. In total, 83% percent of patients had a good performance status. Responses to the previous FOLFOX regimen were as follows: three complete responses (5.6%), 24 partial responses (44.4%), for an overall response rate of 50% (95% CI: 36–64%). The median time to progression and overall survival were 10.3 and 19.2 months, respectively (Recchia *et al*, 2004).

### Response

**Figure 1:**
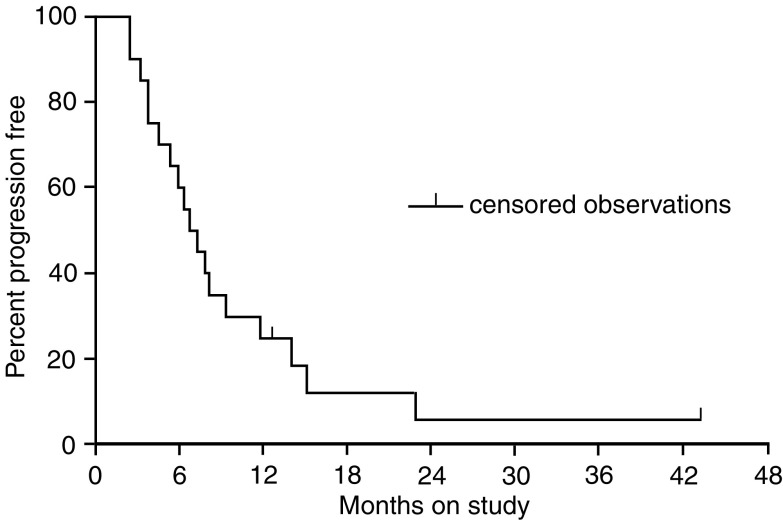
Time to progression. Events 37 (94.2%), censored 2 (5.8%) and median time to progression 7.3 months (range 2.8–43.2 months).

**Figure 2:**
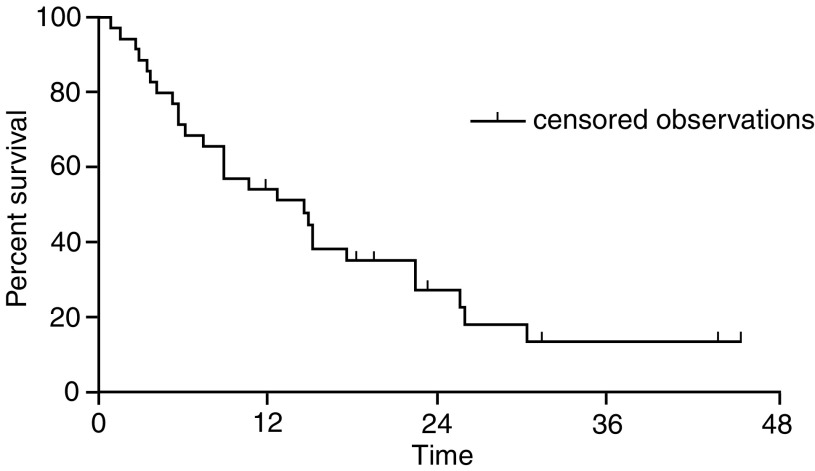
Overall survival. Events 27 (77.1%), censored 8 (22.8%) and median overall survival 14 months (range 1.2–45.6 months).

A total of 251 courses of chemotherapy were administered, with a median number of six courses per patient (range 2–26). All 35 patients were evaluated for toxicity and response. According to the intent-to-treat principle, the following objective remissions were observed: six patients had partial response, giving an overall response rate of 17.1% (95% CI: 6.5–33.6%); 13 patients had stable disease (37.1%) and 16 had disease progression (45.7%). After a median follow-up of 20 months (minimum 12 months), median progression-free survival was 7.3 months (range 2.8–43.2 months) (Figure 1

), while median survival was 14 months (range 1.2–45.6) (Figure 2

). The estimated 1-year survival rate was 55%; however, median survival, calculated from the start of FOLFOX treatment as first-line chemotherapy, was 27 months (range 8.2–59.7 months). Palliative radiotherapy was administered to six patients. The number of instances of disease progression occurring in the following sites was: liver 22, locoregional 8, lung 7, bones 5, brain, peritoneum and nodes, 2 instances each. A total of 25% of patients were salvaged with gemcitabine modulated by 5-FU/LV and with a continuous infusion of 5-FU/carboplatin.

The median DI delivered was 96% for all drugs. The DI of CPT-11 was 84 mg m^−2^ w^−1^, while the DIs of LV and 5-FU were 192 and 960 mg m^−2^ w^−1^, respectively, similar to planned DIs.

### Toxicity

Toxicity data for the 35 patients are summarised in Table 2

**Table 2:** Toxicity according to WHO criteria

	**WHO grade**											
	**0**		**1**		**2**		**3**		**4**		**Total**	
	**No.**	**%**	**No.**	**%**	**No.**	**%**	**No.**	**%**	**No.**	**%**	**No.**	**%**
*Haematologic*												
Leucopenia	12	34	6	17	11	31	4	12	2	6	35	100
Neutropenia	11	31	4	11	7	20	9	27	4	11	35	100
Thrombocytopenia	29	82	2	6	1	3	3	9	0	0	35	100
Anaemia	15	43	13	37	4	11	2	6	1	3	35	100
Infection	26	73	6	18	3	9	0	0	0	0	35	100
												
*Gastrointestinal*												
Oral	22	62	7	20	3	9	2	6	1	3	35	100
Nausea and vomiting	14	40	11	31	7	20	3	9	0	0	35	100
Diarrhoea	14	40	7	20	8	22	6	18	0	0	35	100
Ileus	0	0	0	0	0	0	0	0	0	0	35	100
Hepatic	29	82	6	18	0	0	0	0	0	0	35	100
												
Neurotoxicity	33	94	2	6	0	0	0	0	0	0	35	100
Triglycerides	0	0	0	0	0	0	0	0	0	0	0	0
Renal	35	100	0	0	0	0	0	0	0	0	35	100
												
*Cutaneous*												
Alopecia	0	0	15	44	16	45	4	11	0	0	35	100
Skin	29	83	4	11	2	6	0	0	0	0	35	100

WHO=World Health Organisation.

. No treatment-related death was observed. Grade 3–4 diarrhoea occurred in six patients (18%). Such a low complication rate may be explained by the low daily dose of CPT-11. In fact, a phase I study demonstrated that the gastrointestinal toxicity induced by CPT-11 increased in intensity with greater doses of the drug (Albigerges *et al*, 1995). Leukopenia grade 3–4 occurred in six patients (18%). Grade 3–4 thrombocytopenia was low and was observed in three patients (9%) only. Hepatic toxicity (abnormality of liver enzymes) was observed in six patients (18%); however, two of these patients indulged in alcohol consumption. In all, 40% of patients had no nausea or vomiting. Mild skin toxicity occurred in six patients (17%). Severe alopecia was observed in 11% of patients. Catheter-related complications (displacement, infection) were observed in four patients, all of whom had the catheter removed and a second catheter inserted. Treatment was delayed in 42 courses of chemotherapy (7%).

## DISCUSSION

This multicentre phase II study was designed to assess the activity and toxicity of CPT-11 administered over 2 days, combined with a standard dose of the ‘de Gramont’ regimen in a group of patients with MCC who had been pretreated with the FOLFOX regimen as first-line chemotherapy. The objective response rate was 17.1% (95% CI: 6.5–33.6%) and median progression-free survival was 7.3 months. An overall clinical benefit was observed in 54% of patients. The relatively long median survival of 14 months from the start of the second-line chemotherapy and 27 months from the diagnosis of metastatic disease, that is, from the administration of the FOLFOX regimen as first-line treatment, may be explained by the fact that two patients with a partial response underwent liver resection of residual metastatic disease and are still alive after 43 and 45 months, respectively, and 25% of patients received a third line of chemotherapy with 5-FU modulated by gemcitabine. The administration of a third line of chemotherapy to our patients has been made possible due to the low-toxicity profile resulting from the bifractionated administration of both L-OHP in first-line chemotherapy and campthotecin in second-line chemotherapy. The lower daily dose of CPT-11 decreases the peak plasma level, thus decreasing the toxicity profile, while efficacy is not altered (Albigerges *et al*, 1995). In view of the palliative intents that chemotherapy accomplishes in the treatment of pretreated colorectal cancer, current approaches should be designed to find active but less toxic drug combinations. Palliation of symptoms is important in relatively chemoresistant tumours such as gastrointestinal cancers or non-small-cell lung cancer, in which chemotherapy has no curative intent (O'Connell, 1998). Treatment compliance for this regimen was good, with median relative DIs delivered for L-OHP, LV and 5-FU of 92%, 92% and 94%, respectively.

In the preoxaliplatin era, CPT-11 was shown to be an active agent in patients with rapidly progressing colorectal cancer (Rothenberg *et al*, 1996), in patients refractory to 5-FU (Rougier *et al*, 1998) and in patients who had progressed during or shortly after 5-FU-based chemotherapy (Pitot *et al*, 1997). In Rothenberg's study, however, 23% of patients developed grade 4 diarrhoea and the other studies reported a high-toxicity profile with several hospital deaths attributable to multiple gastrointestinal toxicities, together with unexpected thromboembolic events (Rothenberg *et al*, 2001). The toxicity reported in the American studies was partially due to the different delivery schedule of CPT-11/LV/5-FU, and in a study in which CPT-11 was administered on a weekly basis, diarrhoea was reported in 88.9% of patients (Douillard *et al*, 2000). A recently published randomised phase III study has investigated the efficacy of the alternate sequence of administration of the FOLFIRI and FOLFOX regimens in the treatment of MCC (Tournigand *et al*, 2004). Second-line treatment with FOLFIRI achieved a 4% response rate with a 2.5 month median progression-free survival rate and overall survival of 20.6 months from the start of FOLFOX administered as first-line chemotherapy. Grade 3–4 neutropenia were observed in 21% of patients. In our present study, we report a 20% response rate, a median progression-free survival of 7.1 months and an overall survival of 27 months with grade 3–4 neutropenia observed in 38% of patients. This improvement with respect to relatively poor efficacy of FOLFIRI as second-line therapy reported in Tournigard's study may be due to the fractionated administration of CPT-11, however at the expenses of a slightly worse toxicity profile. The above results show that the efficacy of a chemotherapeutic regimen may be not only dose dependent but also schedule dependent. The administration of CPT-11 over 2 days has been shown to be feasible, active and tolerable and our 1-year survival rate of 56% compares favourably with the 39 and 38% 1-year survival rates obtained in other trials (Rothenberg *et al*, 1999, Gil-Delgado *et al*, 2001) with CPT-11 treatment as second line chemotherapy. As the question of whether fractionated CPT-11 is more effective than CPT-11 given as a single dose on day 1 cannot be assessed from a phase II study, a randomised study comparing fractionated *vs* 1-day administration of CPT-11 with 5FU/LV is shortly planned to clarify this issue.
